# Phyllostomid bat microbiome composition is associated to host phylogeny and feeding strategies

**DOI:** 10.3389/fmicb.2015.00447

**Published:** 2015-05-19

**Authors:** Mario Carrillo-Araujo, Neslihan Taş, Rocio J. Alcántara-Hernández, Osiris Gaona, Jorge E. Schondube, Rodrigo A. Medellín, Janet K. Jansson, Luisa I. Falcón

**Affiliations:** ^1^Laboratorio de Ecología Bacteriana, Instituto de Ecología, Universidad Nacional Autónoma de MéxicoCoyoacán, Mexico; ^2^Earth Sciences Division, Ecology Department, Lawrence Berkeley National LaboratoryBerkeley, CA, USA; ^3^Instituto de Investigaciones en Ecosistemas y Sustentabilidad, Universidad Nacional Autónoma de MéxicoMorelia, Mexico; ^4^Laboratorio de Ecología y Conservación de Vertebrados Terrestres, Instituto de Ecología, Universidad Nacional Autónoma de MéxicoMorelia, Mexico; ^5^Biological Sciences Division, Pacific Northwest National LaboratoryRichland, WA, USA

**Keywords:** microbiome, diversity, Phyllostomidae, feeding-strategies

## Abstract

The members of the Phyllostomidae, the New-World leaf-nosed family of bats, show a remarkable evolutionary diversification of dietary strategies including insectivory, as the ancestral trait, followed by appearance of carnivory and plant-based diets such as nectarivory and frugivory. Here we explore the microbiome composition of different feeding specialists: insectivore *Macrotus waterhousii*, sanguivore *Desmodus rotundus*, nectarivores *Leptonycteris yerbabuenae* and *Glossophaga soricina*, and frugivores *Carollia perspicillata* and *Artibeus jamaicensis*. The V4 region of the 16S rRNA gene from three intestinal regions of three individuals per species was amplified and community composition and structure was analyzed with α and β diversity metrics. Bats with plant-based diets had low diversity microbiomes, whereas the sanguivore *D. rotundus* and insectivore *M. waterhousii* had the most diverse microbiomes. There were no significant differences in microbiome composition between different intestine regions within each individual. Plant-based feeders showed less specificity in their microbiome compositions, whereas animal-based specialists, although more diverse overall, showed a more clustered arrangement of their intestinal bacterial components. The main characteristics defining microbiome composition in phyllostomids were species and feeding strategy. This study shows how differences in feeding strategies contributed to the development of different intestinal microbiomes in Phyllostomidae.

## Introduction

“We are what we eat” is an expression that defines us all, but that is especially represented in the members of the Phyllostomidae, the New World leaf-nosed bat family, that arose at the end of the Eocene (Villalobos and Arita, 2010). Phyllostomids are found from southern USA and northern Mexico to Argentina and are the most ecologically diverse family within the order Chiroptera. They show a remarkable evolutionary diversification of dietary strategies from insectivory as the ancestral trait, to a wide array of diets that include blood, meat from small vertebrates, nectar, fruit and complex omnivorous mixtures (Gardner, 1979). The rise of new lineages in this group is related to the Late Oligocene Warming ~23–26 million years ago (MYA), when the specialization for different diets diversified (Rojas et al., 2011). Present-day patterns of phyllostomid species diversity is a factor of geographic and ecological interactions, with the highest species diversity found in the Amazon basin and the tropical Andes, and the least diversity existing in southern USA, northern Mexico and northern Argentina (Villalobos and Arita, 2010). There are over 190 species within the Phyllostomidae, with *Macrotus* being the most ancient genus appearing around 35 MYA, and characterized by insect-feeding specialization. The next step in the evolution of feeding strategies in this family was described as carnivory and sanguivory, the later including the subfamily Desmodontinae with three genera *Desmodus, Diphylla*, and *Diaemus*. Nectar and fruit-eating species are the most recent (~20–18 MYA) and include the greatest radiation within the family (Rojas et al., 2011).

Phyllostomids are a model clade to test the relationship between microbiome-host composition, phylogeny and the evolution of feeding strategies since this bat family shows species-specific feeding strategy specialization. We know that the microbiome is intimately related to the individuals health, development stage and evolution of diet in mammals, playing a crucial role in nutritional processes in the intestine by complementing the digestive capabilities of the host (Savage, 1977; Bäckhed et al., 2004; Nicholson et al., 2005; Turnbaugh and Gordon, 2009; Lee and Mazmanian, 2010). Coevolution between hosts and their intestinal microbiota is considered a process of mutual adaptations that is key to biological diversification (Brockhurst and Koskella, 2013). So, it has become evident that the adaptive landscape, which represents the relation between an organism's fitness and attributes, is the sum of the host-microbiome super organism association (MacColl, 2011). Studies in humans have shown that intestinal microorganisms have evolved with their hosts and with each other, creating highly organized associations (Van den Abbeele et al., 2011). Archie and Theis (2011) explain that the role of Eubacteria and Archaea within their animal hosts results fundamental in triggering social and genotypic relationships. Bacteria are directly involved in their host's fitness via energy uptake of different food sources, synthesis of vitamins necessary for growth, and are associated to the function of the immune system and the health of their hosts (Nicholson et al., 2005). Gut microbiota is also involved in mate and progeny recognition via odor produced by the microbiome (Lizé et al., 2013). A previous study focusing on the microbiome composition of different members of the order Chiroptera showed that host phylogeny and life history influence microbiome composition (Phillips et al., 2012).

In this study we included several species of phyllostomid bats with different feeding strategies, including *Macrotus waterhousii* (insectivore); *Desmodus rotundus* (sanguivore); *Leptonycteris yerbabuenae* and *Glossophaga soricina* (nectarivores); *Carollia perspicillata* and *Artibeus jamaicensis* (frugivores). All of these species have similar geographic distributions, and share the same habitat and shelter. All individuals were collected in the same cave in southern Mexico, except for *M. waterhousii*, collected in a separate cave, ~400 km away. The main goal of this study was to explore how the microbiome composition of each bat species relates to the feeding-strategy of the host. Several studies have suggested that host phylogeny influences microbiome composition over other factors, including diet and environment (Ochman et al., 2010; Roeselers et al., 2011; Phillips et al., 2012; Sanders et al., 2014), while others argument that diet strongly influences microbiome composition (Muegge et al., 2011). In this study we hypothesize that microbiome composition will converge in relation to both diet and host phylogeny.

## Materials and methods

### Sampling

Bats were captured and handled under permission from the Dirección General de Vida Silvestre, SEMARNAT, Mexico to Jorge E. Schondube (SGPA/DGVS/12889/13). Bats were sampled using mist nets placed at the entrance and inside of two caves in central-southern Mexico, Huarache cave in Palo Blanco, Guerrero (17°32′12″N, 99° 28′ 15″ W) and Vegas cave, located 5 km south of Tenampulco, Puebla (20°08′54″ N, 97°24′39″ W) (Brunet and Medellín, 2001). All species of phyllostomids included in this study were sampled at Vegas cave, except for *M. waterhousii* that was only found at Huarache cave. All captures took place in August 2012. From each bat we recorded: weight, forearm length, age, sex and reproductive condition (Table 1). Due to ethical considerations and permit limitations, we collected three non-reproductive individuals per species except *L. yerbabuenae* of which we collected two individuals. Bats were euthanized with ether, and all efforts were made to minimize suffering following the humane handling guidelines approved by the American Society of Mammalogists (Sikes and Gannon, 2011).

**Table 1:** **Phylogenetic community alpha diversity metrics for microbiome of specialized feeding strategist-phylostomid bats**.

	**Faith's PD**	**Shannon's H'**	**Number of OTUs**	**Fisher's alpha**	**Feeding strategy**	**Age**	**Sex**	**Weight (gr; mean ± SD)**	**Forearm length (mm; mean ± SD)**	**Location cave name**
*A. jamaicensis*	30	4	442	68	Frugivory	Juveniles/Adults	M	29.0 ± 3.6	60.8 ± 0.6	Las Vegas, Puebla, México
*C. perspicillata*	203	7	2963	782	Frugivory	Adult	M	18.0 ± 1.0	44.0 ± 0.8	Las Vegas, Puebla, México
*G. soricina*	42	4	537	86	Nectarivory	Juveniles/Adults	M	10.5 ± 0.5	36.8 ± 0.8	Las Vegas, Puebla, México
*L. yerbabuenae*	133	7	1860	410	Nectarivory	Adult	M	23. 5 ± 0.6	54.7 ± 0.2	Las Vegas, Puebla, México
*D. rotundus*	289	10	4940	1446	Sanguivory	Adult	M + F	29.0 ± 1.0	57.8 ± 1.5	Las Vegas, Puebla, México
*M. waterhousii*	254	10	4343	1215	Insectivory	Adult	M	18.5 + 0.5	52.5 ± 0.6	El Huarache Guerrero, México

### Gut dissection and DNA extraction

The dissection of the gut was made following Nordgård et al. (2005) with some modifications. Given the complex structure and diverse functional roles of the gut (digestion, nutrient recycling, waste production, etc.), we obtained samples from the whole intestine to understand the real dimension of its gut bacterial diversity. The intestinal region was measured and cut in three equal parts, stored with 0.5 ml of DNA extraction buffer (100 mM Tris-HCl, 20 mM NaCl, and 100 mM EDTA, pH = 8) in liquid nitrogen. Since it is not possible to identify each intestinal section (ileum, jejunum, duodenum, and hind gut) without help of stereoscopy, and doing a correct morphological identification takes time and implies conducting several cuts in the tissue, we decided to divide the intestine in three fractions of similar size (anterior, medium, and posterior). By doing so, we reduced the possibility of contamination samples, and limited the changes in the intestinal bacterial composition that occur due to modifications of the intestinal ecosystem that follows tissue death. While the three sections of the intestine we analyzed do not correspond directly with functional regions, they allowed us to describe changes in bacterial diversity along a gradient of intestinal function where digestion and absorption of nutrients decreases, and water absorption and waste management increases toward the anus. All samples were placed in sterile tubes and immediately stored in liquid nitrogen until DNA extraction. Zirconium beads (500 mg), 600 μl saline solution (0.85% NaCl and 0.1% Tween), 60 μl SDS (sodium dodecyl sulfate) and 60 μl CTAB (cetyltrimethylammonium bromide) were added to the tubes, and samples were mechanically disrupted with a bead-beater (FastPrep FP120, Bio101, CA, USA). Samples were decanted and the aqueous phase recovered for lysis (0.2 M NaOH, 2 mg/ml lysozyme and 1% SDS) at 37°C for 90 min. A subsequent lysis step was conducted with an overnight incubation with proteinase K at 50°C (Sigma-Aldrich, CA, USA). An organic solvent extraction based on phenol-chlorophorm-isoamyl alcohol (25:24:1) (Sigma) was applied to all samples and repeated three times. Finally, DNA was precipitated with 1 volume of ice-cold 97% propanol and 0.1 volume of 3 M sodium acetate. The obtained pellets were washed with ethanol 80%, resuspended in 30 μl of molecular grade water and stored at −70° until PCR amplification.

### 16S rRNA gene amplification and sequencing

We followed the protocol described by Caporaso et al. (2012) for paired-end 16S rRNA gene community sequencing using primers 515F/806R that target the hypervariable region V4 in both bacteria and archaea. Intestine regions per individual and species were treated as a separate sample, and each PCR included a specific Golay reverse primer (Caporaso et al., 2010). DNA concentrations were calculated from each sample with a Qubit dsDNA assay (Invitrogen, Carlsbad, CA). On average 2 ng/μl of total DNA were added to each PCR reaction, of a total volume of 25 μl, and had 2.5 μl Takara (TaKaRa Corp., Shiga, Japan) ExTaq PCR 10X buffer, 2 μl Takara dNTP mix (2.5 mM), 0.7 μl bovine serum albumin (20 mg/ ml, Roche), forward and reverse primers (10 mM final concentration), 0.125 μl Takara Ex Taq DNA Polymerase (5u/μl) and nuclease free-water. The amplification protocol included an initial denaturalization step at 95°C for 3 min, followed by 35 cycles of 95°C, 30 s, 52°C, 40 s, 72°C, 90 s, and a final extension at 72°C for 12 min. Each sample was amplified in triplicate, combined and purified using the SPRI magnetic bead, Agencourt AMPure XP PCR purification system (Beckman Coulter, Brea, CA, USA). DNA concentration after pooling the PCR products for each sample and purification steps were obtained with the Qubit dsDNA HS assay. Amplicons were pooled (~20 ng per sample) and sequenced on Illumina MiSeq platform (at the Yale Center for Genome Analysis, CT, USA), resulting in ~250 bp paired end reads. The sequence data are available from BioProject ID: PRJNA260412.

Paired-end sequences were overlapped and merged using FLASH (Magoč and Salzberg, 2011). Quality filtering and demultiplexing were performed in QIIME (*r* = 1; *p* = 0.75; *q* = 3; *n* = 0,–min_count 0.005%) as described previously (Caporaso et al., 2012; Bokulich et al., 2013). For *L. yerbabuenae* two samples did not pass the quality filtering and were excluded from further analysis. Sequences were grouped into operational taxonomic units (OTUs) based on 97% sequence identity, and chimeric sequences were removed, using USEARCH (Edgar, 2013). OTUs were given taxonomic assignments in QIIME (Caporaso et al., 2012) version 1.7.0 using RDP classifier (Wang et al., 2007) and Greengenes database release 13_5. Phylogenetic trees were created using FastTree2 (Price et al., 2010) under QIIME's default parameters and these trees were used for the calculation of α (Shannon's *H'*, Fisher's and Faith's PD) and β diversity (weighted UniFrac distance) metrics. Communities were standardized to a total number of 12,000 sequences per intestine region per individual, or intestine regions were combined into 44,600 sequences per individual within each species. The weighted UniFrac distance matrices were used to visualize microbiome composition within bat species.

### Data analysis

All statistical tests were conducted by using R packages “ade4” (Chessel et al., 2012) and “vegan” in the R statistical environment (Oksanen et al., 2007). Results are defined to be significant at *P* < 0.05. Correlation amongst bat species, intestinal regions and changes in microbial community abundances were explored via canonical correlation analysis as implemented in ade4. To test differences between α diversity of different bat species with different feeding strategies, we used One-Way ANOVA followed by the Tukey's honestly significant difference test. Ordination of the whole community detected by 16S rRNA gene amplicon sequencing was created from UniFrac matrix calculated by QIIME software and presented in a principal coordinates analysis plot. The contribution of feeding strategy, host species, age, sampling site and sex to β-diversity was tested via permutational MANOVA model as implemented in the “adonis” function of the vegan package in R. For this analysis first each parameter was sequentially added to the model. Secondly, group variations were controlled amongst feeding strategies and bat species. The evolutionary relationships among Phyllostomidae included in this study were inferred from mitochondrial CytB sequence identities. CytB sequences were aligned with Muscle (Edgar, 2004) and the calculated pairwise distances were used for clustering by UPGMA.

## Results

### Microbiome composition of phyllostomid bats

**Figure 1:**
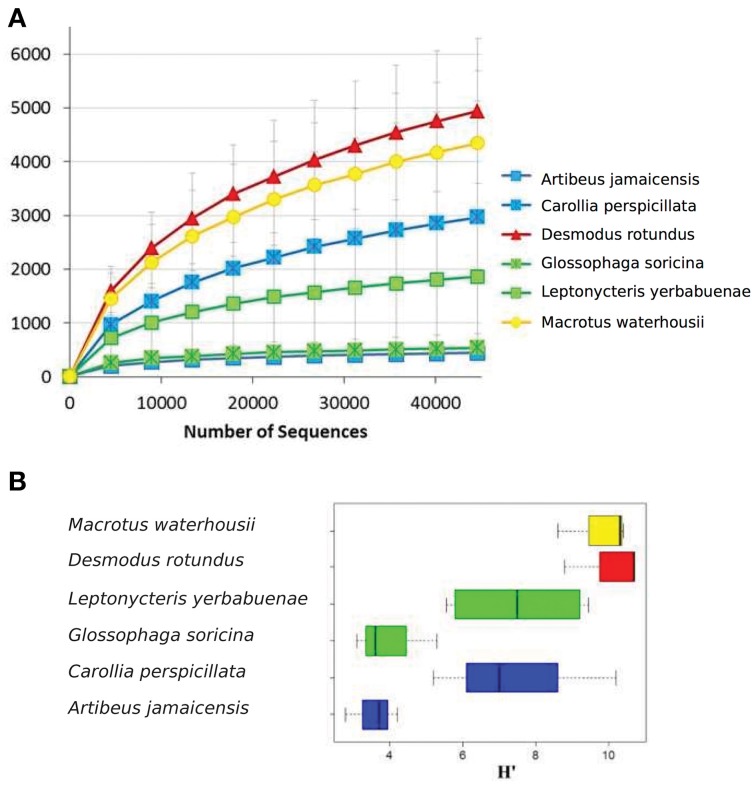
**(A)** Rarefaction curves for OTUs defined at 97% similarity per bat specie. **(B)** Shannon diversity index for microbiome genetic composition based on 16S rRNA gene sequences. In both figures, error bars represent the standard variation observed between different individuals. Colors represent feeding strategies where blue-fruit eaters, green-nectar eaters, red-sanguivore and yellow-insect eater.

A total of 2, 877, 215 16S rRNA gene sequences were obtained for the microbiome of the six species (3 individuals, per species except *L. yerbabuenae* of which we had 2 individuals) of phyllostomid bats included in this analysis: frugivores *A. jamaicensis* and *C. perspicillata;* nectarivores *G. soricina* and *L. yerbabuenae*; the sanguivore *D. rotundus* and the insectivore *M. waterhousii*. After conducting a rarefaction analysis to the same level of surveying effort (12,000 sequences per intestine region within each individual; 44,600 sequences per individual within each species), we found that the observed operational taxonomic units (OTUs) varied widely among species, with a difference that reached an order of magnitude between samples (Figure 1A, Figure S1). The least diverse microbiome (442 OTUs) was found in *A. jamaicensis* -one of the fruit-eating species-, while the sanguivore *D. rotundus* had the most diverse microbiome (4940 OTUs) (Figure 1A, Table 1). In the present study, all diets were equally represented, and a rarefaction analysis was conducted to avoid biasing our dataset. Bacterial α-diversity was lowest in the plant-eater species (fruit and nectar) in comparison to species with diets that included protein and lipid rich elements like insects and blood (Figure 1B, Table 1). It should be noted that α-diversity was highly variable between the different species of fruit and nectar feeding strategies where *A. jamaicensis* and *G. soricina* had the lowest species richness in the data set (Figure 1A, Table 1).

**Figure 2:**
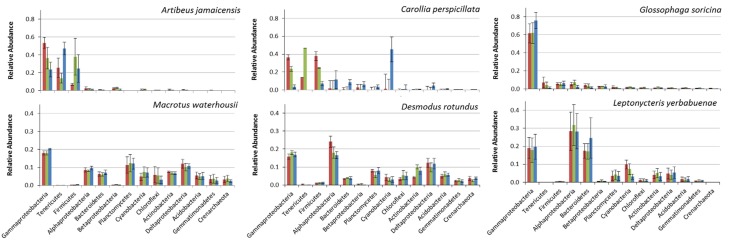
**Comparison of bacterial phyla from three intestinal regions (anterior, red; medium, green and posterior, blue), for fruit (*A. jamaicensis, C. perspicillata*), nectar (*G. soricina, L. yerbabuenae*), blood (*D. rotundus*), and insect-feeding (*M. waterhousii*) phyllostomids**. For each intestinal región, relative abundance of each phylum from different individuals were averaged (*n* = 3, except *L. yerbabuenae n* = 2, values are reported in Table S1).

**Figure 3:**
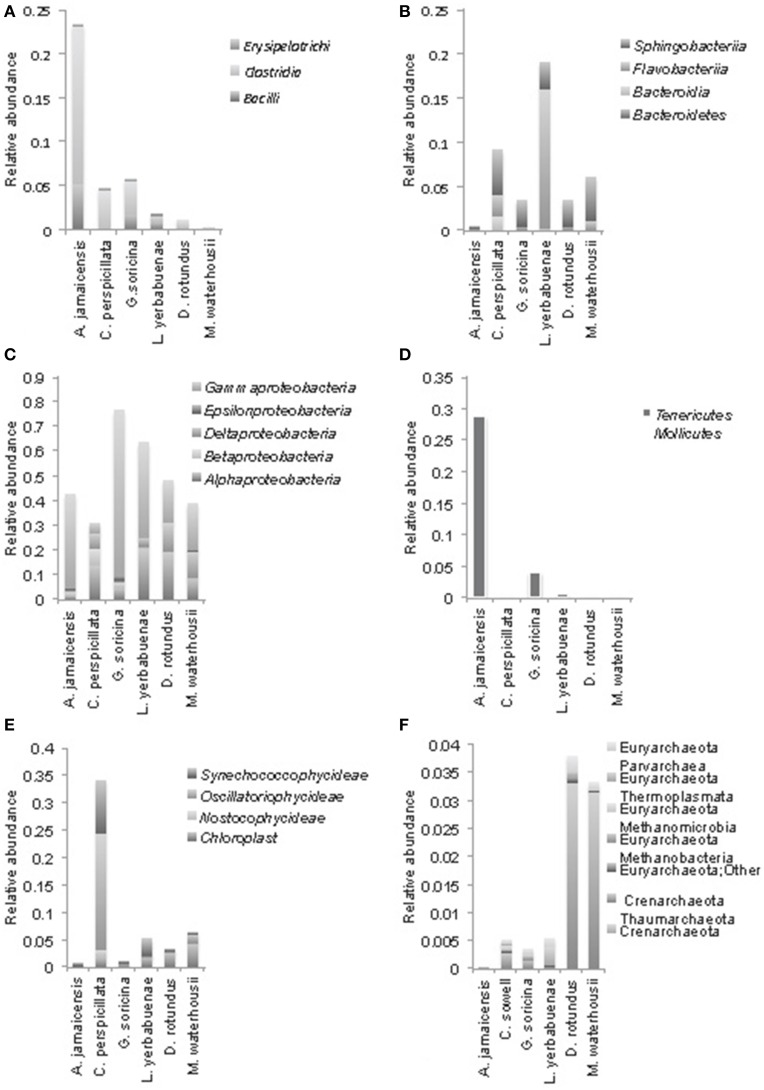
**Phylogenetic composition of the most abundant bacterial phyla at class level in microbiomes of phyllostomid bats. (A)** Firmicutes, **(B)** Bacteroidetes, **(C)** Proteobacteria (Gamma, Epsilon, Delta, Beta, and Alpha); **(D)** Tenericutes, Mollicutes; **(E)** Cyanobacteria; **(F)** shows Archaea composition. In each bat species, class level relative abundances were averaged from total gut compositions (sum of anterior, medium, and posterior) for each individual bat.

Amongst different bat feeding strategies, we detected host-specific differences in bacterial community composition (Figure 2). The bacterial phyla that contributed most to differences in microbiomes between bat species were: Gamma-, Alpha-, and Delta-proteobacteria, Tenericutes, Firmicutes, Bacteroidetes, Planctomycetes, and Cyanobacteria, the last of which were only found in relevant numbers in the first gut section of the fruit-feeder *C. perspicillata* (Figures 2, 3 and Figure S2). Overall, the differences observed in microbiome composition among different intestinal regions of bat species were only marginal (Figure 2). However, relative abundance of major gut phyla of fruit eating species, *A. jamaicensis* and *C. perspicillata*, were different between three intestinal regions (Figure 2). We explored relationships between microbial phylum level relative abundances, bat species and their intestinal regions via canonical correspondence analysis. In *A. jamaicensis* (frugivore) Tenericutes and Firmicutes populations had a strong positive correlation with medium and posterior sections of the intestine (Figure S2) whereas the same intestinal regions of the other frugivore, *C. perspicillata* showed the strongest correlation between Betaproteobacteria and Bacteriodetes.

Proteobacteria were the most abundant bacterial phyla in all of the feeding strategies analyzed (Figure 3). Gammaproteobacteria were the most abundant group of bacteria in *A. jamaicensis* (frugivore) and *G. soricina* (nectarivore); both bat species had predominantly *Enterobacteriales*, with a large component of *Escherichia* spp. (75–33%, respectively), followed by *Pasteurella*, both common components of animal intestinal microbiota. Deltaproteobacteria showed the reverse trend, representing the smallest proportion of bacteria in *A. jamaicensis* and *G. soricina* compared to the rest of the species included in this study, which had similar trends in composition of *Desulfurellales, Syntrophobacterales*, and *Myxococcales* being the most abundant. The nectar eating *L. yerbabuenae* had the largest amount of Alphaproteobacteria, and all bat species had *Rhodospirillales, Rhodobacterales, Rhizobiales*, and *Rickettsiales* as a common feature, the last of which is a group of bacteria adapted to live within animal host cells, and are thus common in mammal microbiome studies (Philippot et al., 2010).

We observed Firmicutes in all the diets analyzed, with a major composition of *Clostridia* and *Bacilli*. *A. jamaicensis* had the lowest microbiome diversity and was also different in composition showing more Tenericutes (*Mollicutes*) and Firmicutes (*Clostridia* and *Bacillus*) than any other phyllostomid species (Figures 2, 3). The rest of the plant-based feeders had similar trends in microbiome composition except for Cyanobacteria found in the first intestinal region of *C. perspicillata*. Most Cyanobacteria found in the microbiomes related to Group II pseudo-filamentous *Pleurocapsales*, and Group I, unicellular Cyanobacteria (Rippka, 1988), which can be found forming biofilms over moist surfaces, and are common in rainforest environments and cave entrances (Figure 3). This is the second study of bat microbiomes that identifies Cyanobacteria as a component of different bat species (Phillips et al., 2012). *Planctomycetia* were the most abundant Planctomycetes in all of the feeding strategies, except for *A. jamaicensis*, that had remarkably low abundances of this bacterial taxon. These are mainly aerobic and mesophilic organisms that are starting to be reported from a grand variety of environments, including aquatic, terrestrial and extreme environments (Lage and Bondoso, 2014).

The microbiome diversity within *D. rotundus* and *M. waterhousii* were the highest among bat species of this study. Both species have high N content in their diet as evidenced in previous work using stable isotopes (Schondube et al., 2001). *Macrotus*, while feeding mostly on insects, tend to include also fruits in their diet, showing at least part of the year an omnivorous diet (Herrera-Montalvo et al., 2013). This varied diet could be the cause of the high microbiome diversity found in this species. Another striking difference in microbiome composition between plant- (fruit and nectar) vs. animal-eating bats (insectivores and sanguivores) is the great abundance of Crenarchaeota in the later (Figure 3). Overall, archaea are more abundant in the insect and blood eating bats. The presence of archaea in the microbiomes of vertebrates has been recorded in the past, although their role in the intestinal ecosystem is yet to be discovered.

**Figure 4:**
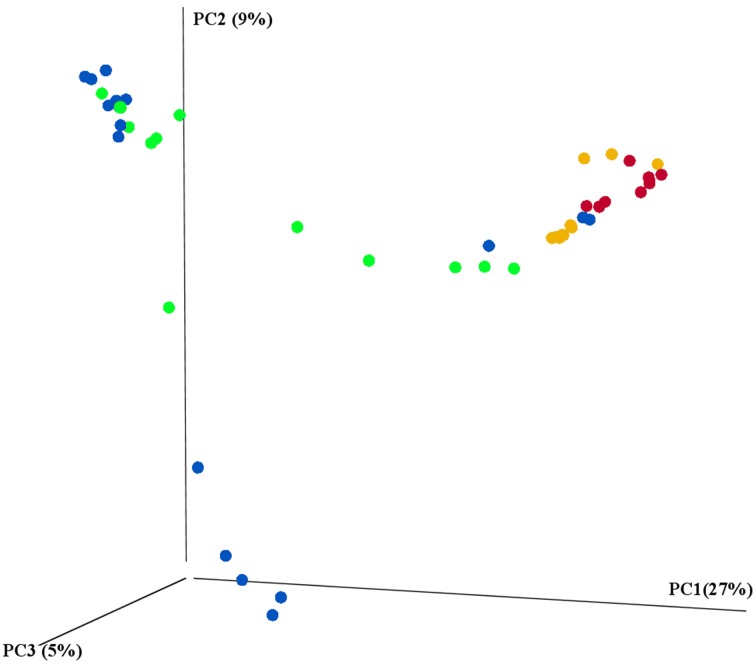
**16S rRNA gene sequencing revealed the differences between prokaryotic diversity of animal-based and plant-based strategists**. Samples were clustered using principal coordinates (PC) analysis of the weighted UniFrac distance matrix. Each circle represents an individual intestinal region per bat where feeding strategies were colored to represent: blue-fruit eaters, green-nectar eaters, red-sanguivore and yellow-insect eater. Significant contributions of bat-traits to observed differences in prokaryotic diversity was tested using adonis and reported in Table 2.

**Figure 5:**
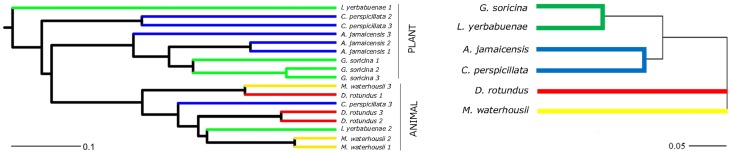
**Comparison between the phylogeny of Phyllostomidae based on mitochondrial CytB sequence identity and clustering of gut microbiomes of Phyllostomidae based on Unifrac distances**. In both trees, branches were colored to highlight bat feeding strategies: blue-fruit eaters, green-nectar eaters, red-sanguivore and yellow-insect eater. UPGMA trees based on the weighted UniFrac distances for among each bat (OTU's from different intestine regions were combined per individual) and overall Phyllostomidae phylogeny showed a grouping within plant- and animal-based diets. Scale bars represent 10% dissimilarity for gut microbiomes and 5% dissimilarity for Phyllostomidae species.

The main characteristics defining microbiome composition identified in this study (Figure 4, Table 2) were bat species (*F* = 14.188, *p* = 0.001) and feeding type (*F* = 11.268, *p* = 0.001), explaining altogether 36% of variation in bacterial phyla composition, and suggesting that diet and host phylogeny are the main drivers of phyllostomid gut microbiota. When controlled for the within group variances between feeding strategies or bat species, these two parameters continued to have the highest contribution to the observed differences in gut microbial composition (Table 2). Bat species with animal-based diets had the most diverse microbiomes when compared both to fruit- and nectar- eating species. Plant and animal specialists clustered within feeding-types, although an individual of the fruit specialist *C. perspicillata* and the nectar-feeder *L. yerbabuenae* clustered with the animal-eating species (Figure 5). It is well documented that plant-feeding strategists eat insects (Fleming, 1988). While they can accidentally ingest insects that are within the flower structures or fruits they feed on most of the time, they also tend to actively hunt for insects at least during some time of the year when their N requirements are higher (Fleming, 1988). Altogether, feeding source was the most relevant feature explaining variation in microbiome composition, and the overall dendogram for the total microbial diversity recovered in all microbiomes follows the phylogenetic structure of the Phyllostomidae (Figure 5).

**Table 2:** **Contribution of bat traits and feeding strategies to observed β-diversity of bat gut microbial communities**.

	***Df***	**Sums of Sqs**	**F. Model**	***r*^2^**	***p***
Bat species	5	1.767	14.188	0.174	0.001^***^
Feeding strategy	3	1.403	11.268	0.138	0.001^***^
Sampling location	1	0.839	4.416	0.083	0.004^***^
Species sex	1	0.318	1.583	0.031	0.130
Intestine section	2	0.274	0.666	0.027	0.758
Species age	1	0.070	0.812	0.007	0.526
Residuals	37	5.474		0.540	
Total	50	10.145		1.000	
**CONTROLLING FOR WITHIN GROUP VARIANCES IN BAT FEEDING STRATEGIES**					
Bat species	5	7.783	8.125	0.489	0.001^***^
Sampling location	1	0.223	1.166	0.014	0.001^***^
Species sex	1	0.415	1.415	0.089	0.572
Intestine section	2	0.280	0.731	0.018	0.831
Species age	1	0.359	1.873	0.023	0.168
Residuals	40	6.864		0.368	
Total	50	15.924		1.000	
**CONTROLLING FOR WITHIN GROUP VARIANCES IN EACH BAT SPECIE**					
Feeding strategy	3	4.365	6.110	0.274	0.021^**^
Sampling location	1	1.415	4.487	0.089	1.000
Species sex	1	0.223	0.938	0.014	0.281
Intestine section	2	0.276	0.580	0.017	0.904
Species age	1	1.536	6.452	0.096	0.277
Residuals	42	8.109		0.509	
Total	50	15.924		1.000	

** P ≤ 0.05,

*** *P ≤ 0.001*.

## Discussion

In this study we explored the correlations among different bat feeding strategies and host microbiome composition. Strikingly our results suggest a strong nested correlation where both host phylogeny and feeding strategy were strong indicators of bat gut microbiome composition. Especially, the higher diversity observed in the microbiome associated to animal-based diets, represents a deviance from the theory that in the mammalian gut, bacterial diversity increases as host diet diverges from carnivorous to omnivorous to herbivorous (Ley et al., 2008). However, in this study, plant-based feeding strategies included nectar and fruit eating bats where food source is broken into simple sugars (Karasov et al., 2011; Herbst and Gables, 2013). In herbivorous animals, digestive system is required to process (hemi)celluloses, lignin-derivatives and insoluble starches which all require multiple enzymes originating from different species thus supporting a highly diverse ecosystem (Karasov et al., 2011). Moreover several recent studies showed that host phylogeny can prevail over diet and environmental factors and can profoundly impact gut microbial diversity (Ochman et al., 2010; Roeselers et al., 2011; Sanders et al., 2014). Even though the reasons behind higher microbial diversity observed in insectivore and sanguivore bats remain unresolved, selective pressures alongside the feeding strategy might be governing the microbial community in these bats. We hypothesize that the high bacterial diversity could be associated to the presence of a diet rich in proteins, lipids and a high concentration of nutrients thus supporting diversity and creating a hotspot for bacterial growth.

Besides low microbial diversity, plant-eating bats also showed large dissimilarities in their microbiome composition, whereas animal-feeding bats showed a clustering effect among members of each feeding strategy (Figure 4). This could be related to the high variation in diet composition that occurs among both fruit- and nectar-eating phyllostomid bats, that tend to include insects, pollen and a large diversity of fruits with different nutritional contents (Klite, 1965; Fleming, 1988; Herbst and Gables, 2013). While different fruits could contain a large variation in nutritional content (Johnson et al., 1985), animal tissues tend to be nutritionally similar, presenting homogeneous composition (Del Rio and Wolf, 2005). Therefore, it can be hypothesized that insectivore and sanguivore bats have more specialized, though diverse, gut microbiome. However, the current study included only one species per animal-based diet (sanguivorous and insectivorous), and thus cannot account for population variations.

Results here presented contrast with the work of Phillips et al. (2012) that suggests an increase in microbiome diversity from sanguivorous, insectivorous, nectarivorous to frugivorous species. Nonetheless, same authors propose that microbiome diversity will be the most diverse in most ancient lineages, thus, for the case of phyllostomids, insectivores and sanguivores would be expected to have the most diverse microbiomes, just as suggested in this study. The previous analysis of microbiome in Chiroptera (Phillips et al., 2012) included mostly frugivorous bats (70%, *n* = 28, of sampled bats) and insectivorous dietS represented by few specimens. The present study is based on a different sequencing technology, and all microbiomes analyzed reach an asymptote for OTU diversity per sample (Figure S1), thus obtaining higher diversity indexes than previous work.

Bacterial phyla that have been associated to diets containing fermentable carbohydrates include Firmicutes and Tenericutes. Interestingly, the fruit-feeder *A. jamaicensis* was the only phyllostomid to show a large Firmicutes: Bacteroidetes ratio (~55%), whereas the rest had ratios between 0.05 and 2%. Also, *A. jamaicensis*, was the largest bat species included in this study. So far it is not understood what the relationship between Firmicutes to Bacteroidetes implies for the ecology and physiology of wildlife. It would be very important to understand the implications of these bacteria to the metabolism of species with a high carbohydrate intake such as *A. jamaicensis*. *Mollicutes* have been reported to flourish on diets rich on carbohydrates, especially associated to fructose and mannose metabolism pathways involved in fermentation of sugar molecules (Arora and Sharma, 2011), and were mostly present in *A. jamaicensis*. This is consistent with observations that fructose is one of the most abundant sugars present in the fruits ingested by this and other fruit-eating phyllostomid bats (Baker et al., 1998). Further, there was presence of chloroplasts and Cyanobacteria in the microbiome of all phyllostomid species, such as reported before for different Chiroptera (Phillips et al., 2012). Behavioral studies on sanguivorous bats have shown that they predominantly feed on blood of large herbivores, and lick their coats before sucking, thus this could be the mechanism for chloroplast acquisition. This behavior has been associated to the origin of blood-sucking, which could have originated from bats licking the wounds of large animals (Fenton, 1992).

This study shows that Phyllostomidae gut microbiome composition is intimately related to host-phylogeny, while feeding-strategy also plays a significant role. Even though we did not find a core phylogenetic Phyllostomidae gut microbiome, within the feeding strategies there might be a core microbiome. Studies in bat gut microbiome are in their infancy and suffer from under sampling and lack of metadata relating to bat life styles, which might have a larger impact on gut microbiome than previously thought. As the only flying mammals, bats are unique model organisms to study impact of host life style and environmental factors on gut development.

## Author contributions

MC, OG, JS, RM, and LF conceived the research. MC, RA, OG, NT, and LF processed samples and obtained results. NT, MC, JJ, and LF analyzed data. All authors contributed on writing the article. LF and JKJ obtained funding for this research.

### Conflict of interest statement

The authors declare that the research was conducted in the absence of any commercial or financial relationships that could be construed as a potential conflict of interest.
