# Supplementary material for: Phyllostomid bat microbiome composition is associated to host phylogeny and feeding strategies
Source: Front Microbiol. 2015 May 19;6:447. doi: 10.3389/fmicb.2015.00447 (PMC4437186; doi:10.3389/fmicb.2015.00447)
Supplement: Supplementary file 1 [file Table1.DOCX]

Supplementary Material

Table S1. Relative abundances of most abundant phyla in three different intestinal regions. Table represents the average values calculated from different individuals (n=3, graphed in Figure 2) and their standard deviations.
