# Supplementary material for: Phyllostomid bat microbiome composition is associated to host phylogeny and feeding strategies
Source: Front Microbiol. 2015 May 19;6:447. doi: 10.3389/fmicb.2015.00447 (PMC4437186; doi:10.3389/fmicb.2015.00447)
Supplement: Supplementary file 2 [file Image1.PDF]

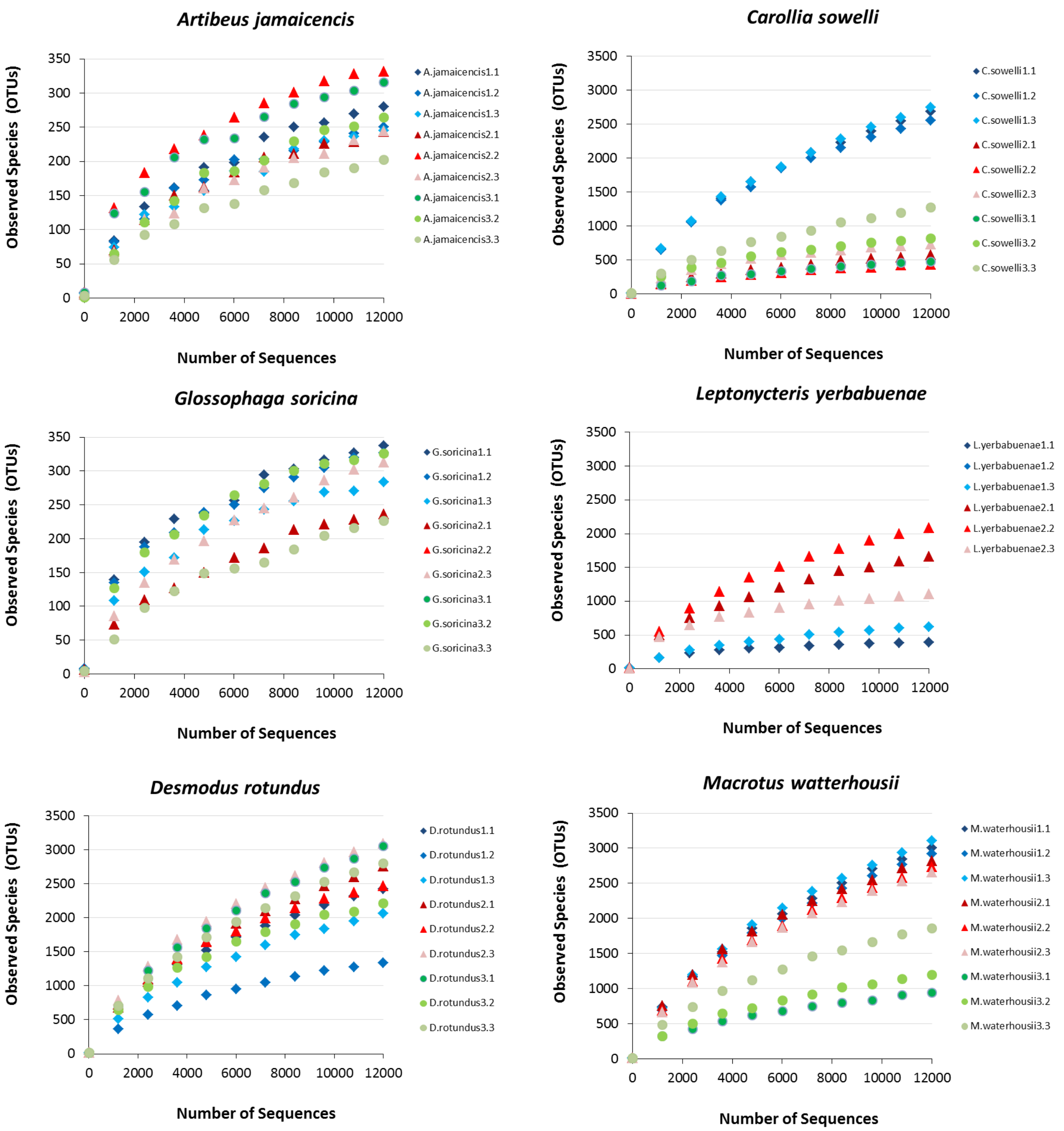

Figure S1. Rarefaction curves indicating observed operational taxonomic units per species analyzed.
