# Supplementary material for: Phyllostomid bat microbiome composition is associated to host phylogeny and feeding strategies
Source: Front Microbiol. 2015 May 19;6:447. doi: 10.3389/fmicb.2015.00447 (PMC4437186; doi:10.3389/fmicb.2015.00447)
Supplement: Supplementary file 3 [file Image2.PDF]

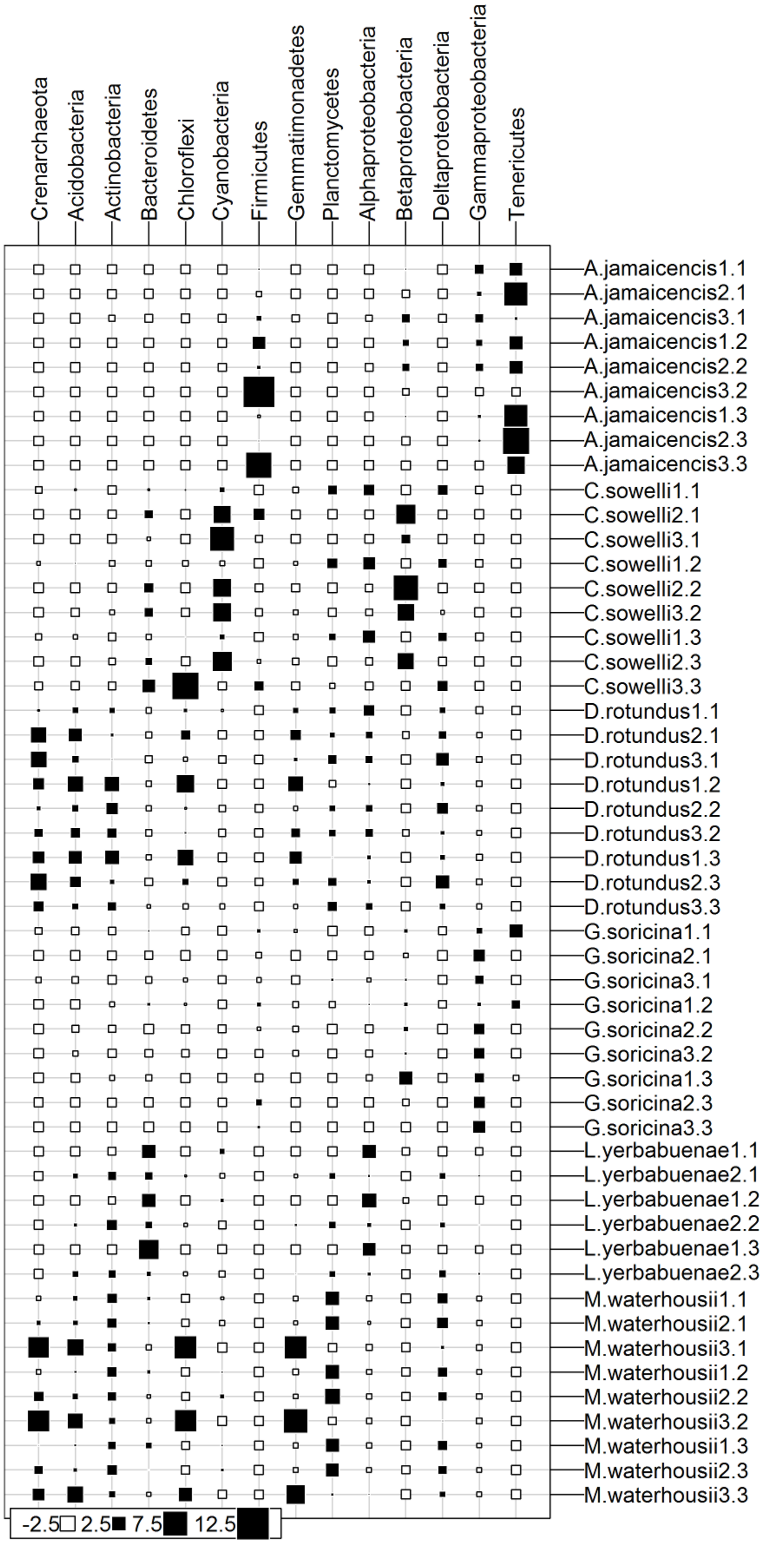
Crenarchaeota
Acidobacteria
Actinobacteria
Bacteroidetes
Chloroflexi
Cyanobacteria
Firmicutes
Gemmatimonadetes
Planctomycetes
Alphaproteobacteria
Betaproteobacteria
Deltaproteobacteria
Gammaproteobacteria
Tenericutes
A.jamaicencis1.1
A.jamaicencis2.1
A.jamaicencis3.1
A.jamaicencis1.2
A.jamaicencis2.2
A.jamaicencis3.2
A.jamaicencis1.3
A.jamaicencis2.3
A.jamaicencis3.3
C.sowelli1.1
C.sowelli2.1
C.sowelli3.1
C.sowelli1.2
C.sowelli2.2
C.sowelli3.2
C.sowelli1.3
C.sowelli2.3
C.sowelli3.3
D.rotundus1.1
D.rotundus2.1
D.rotundus3.1
D.rotundus1.2
D.rotundus2.2
D.rotundus3.2
D.rotundus1.3
D.rotundus2.3
D.rotundus3.3
G.soricina1.1
G.soricina2.1
G.soricina3.1
G.soricina1.2
G.soricina2.2
G.soricina3.2
G.soricina1.3
G.soricina2.3
G.soricina3.3
L.yerbabuenae1.1
L.yerbabuenae2.1
L.yerbabuenae1.2
L.yerbabuenae2.2
L.yerbabuenae1.3
L.yerbabuenae2.3
M.waterhousii1.1
M.waterhousii2.1
M.waterhousii3.1
M.waterhousii1.2
M.waterhousii2.2
M.waterhousii3.2
M.waterhousii1.3
M.waterhousii2.3
M.waterhousii3.3
-2.5 2.5 7.5 12.5

Figure S2. Relationships between phyla abundances, bat species and their intestinal regions were explored via canonical correspondence analysis. The color scale indicates that negative correlation values are in white, whereas positive correlation values are black. Size of the squares designates the strength of the correlation. Rows represent each individual bat as: Species name, Intestinal region (1-Anterior, 2-Medium, 3- Posterior), individual bat (n=2 for *L. yerbabuenae*, n=3 for all the rest).
